# The ameliorative effects of quercetin and curcumin against subacute nephrotoxicity of fipronil induced in Wistar rats

**DOI:** 10.1093/toxres/tfad034

**Published:** 2023-05-22

**Authors:** Meltem Uzunhisarcikli, Fatma Gokce Apaydin, Hatice Bas, Yusuf Kalender

**Affiliations:** Vocational High School of Health Services, Gazi University, Ankara 06830, Türkiye; Faculty of Science, Department of Biology, Gazi University, Ankara 06500, Türkiye; Faculty of Arts and Science, Department of Biology, Bozok University, Yozgat 66100, Türkiye; Faculty of Science, Department of Biology, Gazi University, Ankara 06500, Türkiye

**Keywords:** fipronil, quercetin, curcumin, kidney, histopathology, oxidative stress

## Abstract

Fipronil is a phenylpyrazole insecticide that is widely used in agricultural, veterinary, and public health fields for controlling a wide variety of insect species and it is an environmentally potent toxic substance. Curcumin and quercetin, which are well-known natural antioxidants, are widely used to prevent the harmful effects of free radicals on biological systems. The present study aimed to determine the potential ameliorative effects of quercetin and/or curcumin on fipronil-induced nephrotoxicity in rats. Curcumin (100 mg/kg of body weight), quercetin (50 mg/kg of body weight), and fipronil (3.88 mg/kg of body weight) were administered to male rats by intragastric gavage for 28 consecutive days. In the present study, body weight, kidney weight, the renal function markers (blood urea nitrogen, creatinine, and uric acid levels) in the blood, antioxidant enzyme activities, and malondialdehyde level as markers of oxidative stress, and histological changes of the renal tissue were evaluated. The levels of serum blood urea nitrogen, creatinine, and uric acid were significantly increased in fipronil-treated animals. Additionally, while superoxide dismutase, catalase, glutathione-S-transferase, and glutathione peroxidase activities were decreased in the kidney tissue of rats treated with fipronil, malondialdehyde level was significantly increased. Histopathological analyses showed that the glomerular and tubular injury occurred in the renal tissue of fipronil-treated animals. Also, the supplementation of quercetin and/or curcumin with fipronil significantly improved fipronil-induced alterations in renal function markers, antioxidant enzyme activities, malondialdehyde levels, and histological features of renal tissue.

## Introduction

Because of the intensive use of pesticides for veterinary, agricultural, and various public health purposes, exposure to pesticide residues in food products or the environment is a global public health problem, especially in developing countries. The widespread use of pesticides threatens not only humans but also all nontarget species and causes serious concerns.

Fipronil is a phenylpyrazole group pesticide widely used throughout the world. Additionally, fipronil acts on both target and nontarget organisms, including humans.^1^^,^^2^ Fipronil has various uses in agricultural fields, including veterinary and household applications to control flies, ants, rootworms, weevils, termites, wasps, insects, cockroaches, ticks, lice, mole crickets, fleas and we can say others. It has a long half-life; this means that it has long-half life in the environment.^1^^,^^3^^,^^4^ Because of its lipophilic property, it can accumulate in tissues with high lipid content.^5^ It has been reported that fipronil caused alterations in several biochemical parameters and kidney and brain tissue antioxidant enzymes in mice^3^ and caused endocrine system distribution,^6^ oxidative damage, and histological changes in liver tissue in rats.^7^^,^^8^

Recently, many compounds taken in the diet have been isolated and started to be used in toxicity studies because of their therapeutic properties.^9^ Flavonoids are compounds produced in plants as secondary metabolites. It has many protective biological activities, such as anti-oxidative,^10^^,^^11^ anti-inflammatory, and anti-apoptotic effects.^12^ Phenolic compounds can remove toxic compounds in various organ systems including kidney.^13^ Many investigations have shown that quercetin and curcumin have healing effects on organ toxicity such as reproductive toxicity,^10^ heart toxicity,^14^ and brain toxicity.^15^

Quercetin is a polyphenolic compound used in pathological conditions related to inflammation, viral, or cardiovascular diseases, especially because of its anti-oxidative protective effect. Quercetin is found in various fruits and vegetables such as red apples, green tea strawberries, red wine, and red onions.^16^ Although it can have this protective effect with various mechanisms, it generally makes this protective effect with the free radical scavenging properties.^16^^,^^17^

Curcumin is a polyphenolic compound obtained from the rhizomes of yellow-colored turmeric with lipophilic properties.^9^ It has many therapeutic effects like anti-inflammatory properties,^18^ cytoprotective effects,^19^ and DNA-protective properties.^20^

Based on this information, it is critical to investigate the relationship between fipronil-induced poisoning and phenolic agents with this effect. This study aimed to provide to report that fipronil treatment causes changes in renal damage markers, antioxidant status changes, kidney histopathology, and to investigate the interaction of quercetin and curcumin with this effect.

## Materials and methods

### Chemicals and reagents

Fipronil (PESTANAL, analytical standard; product code: 46451; empirical formula: C_12_H_4_Cl_2_F_6_N_4_OS; molecular weight: 437.15 g/mol; CAS number: 120068-37-3; ≥98.8% purity), quercetin (product code: 337951; molecular weight: 302.21 g/mol; CAS number: 849061-97-8; ≥95% purity), and curcumin [curcumin from *Curcuma longa* (Turmeric), powder; product code: C1386; molecular weight: 368.38 g/mol; CAS number. 458-37-7; assay ≥65% (HPLC)] were purchased from Sigma Aldrich (Germany) and used.

DL-Dithiothreitol, 5,5’-Dithiobis (2-nitrobenzoic acid) (DTNB), ethylenediaminetetraacetic acid disodium salt dihydrate (EDTA), phenylmethylsulfonyl fluoride, potassium chloride, hydrogen peroxide solution, Triton X-100, Trizma base, pyrogallol, L-glutathion reduced, β-Nicotinamide adenine dinucleotide 2′-phosphate reduced tetrasodium salt hydrate (β-NADPH), glutathion reductase, ammonium sulfate, trichloroacetic acid, 1-chloro-2,4-dinitrobenzene (CDNB), butylated hydroxytoluene, 2-thiobarbituric acid (TBA), bovine serum albumin, and disodium hydrogen phosphate were obtained from Sigma Aldrich. Hydrochloric acid, Folin–Ciocalteu’s phenol reagent, sodium hydroxide, and potassium dihydrogen phosphate were purchased from Merck (Germany).

### Animals and experimental protocol

Forty-two adult male Wistar albino rats (250–300 g) were purchased from Gazi University Laboratory Animals Raising and Experimental Research Center. The rats in the plastic cage were housed for 10 days to be acclimatized before entering the experimental study under standardized conditions (temperature: 20 ± 2°C; humidity: 40–45% and 12-h light/dark period). During the experimental period, rats were fed ad libitum standard pellet rat chow and drinking water. All experiments using animals were performed with the approval of the Gazi University Animal Experiments Local Ethics Committee (G.U. ET-18.100).

Forty-two rats were randomly assigned to 7 groups (6 rats/group) as follows:

*Control Group:* the rats were given orally 1 ml/kg body weight (bw) corn oil by gastric gavage for 28 days.

*Quercetin Group:* the rats were given quercetin (50 mg/kg bw daily) in corn oil by gastric gavage for 28 days.

*Curcumin Group:* the rats were given curcumin (100 mg/kg bw daily) in corn oil by gastric gavage for 28 days.

*Fipronil Group:* the rats were given fipronil (3.88 mg/kg bw daily) in corn oil by gastric gavage for 28 days.

*Fipronil plus Quercetin Group*: the rats were given quercetin (50 mg/kg bw daily in corn oil) and fipronil (3.88 mg/kg bw daily corn oil) by gastric gavage for 28 days, respectively.

*Fipronil plus Curcumin Group:* the rats were given curcumin (100 mg/kg bw daily in corn oil) and fipronil (3.88 mg/kg bw daily in corn oil) by gastric gavage for 28 days, respectively.

*Fipronil plus Quercetin plus Curcumin Group*: the rats were given quercetin (50 mg/kg bw daily), curcumin (100 mg/kg bw daily in corn oil), and fipronil (3.88 mg/kg bw daily in corn oil) by gastric gavage for 28 days, respectively.

**Fig. 1:**
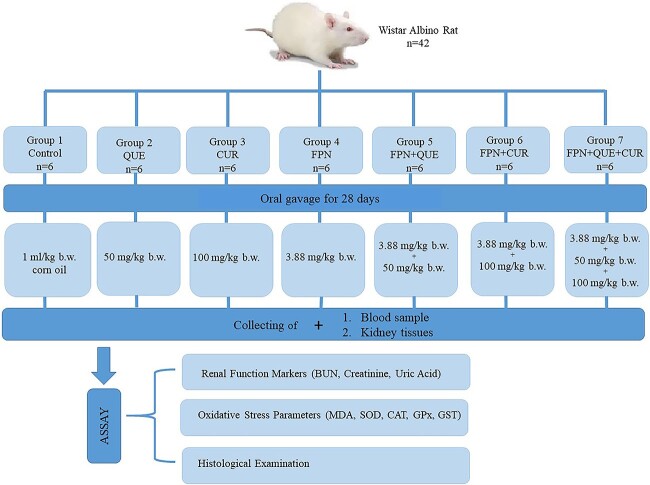
Experimental protocol. *FPN Fipronil; QUE Quercetin; CUR Curcumin.*

Corn oil was used as a vehicle for a dissolving of the experimental compounds. Corn oil treatment alone was defined as the vehicle control group. All tested compounds were administered via gastric intubation to non-fasted animals in the morning (from 09:00 to 10:00) hour for 7 days per week for 4 weeks. The details of the treatment schedule are given in Fig. 1.

The dose of fipronil was selected 1/25 of LD_50_ based on oral LD_50_ of fipronil (97 mg kg^−1^ bw) in rats.^21^ The doses of quercetin and curcumin were detected on the basis of the doses reported to be most effective in reducing toxicity induced by environmental contaminants.^22–24^

At the end of the experimental period (on day 29), all rats were anesthetized through intramuscular injection of xylazine–ketamine combination. Blood samples were obtained from the heart into non-heparinized tubes and then the serum was separated by centrifuging the blood at 3,500 rpm for 20 min for renal function tests. The 2 kidneys of each rat were quickly removed, 1 kidney was quickly frozen in liquid nitrogen (snap freezing) and stored at −80°C until analysis of oxidative stress biomarkers. The other one was preserved with 10% formaldehyde for histological analysis with H&E staining.

### Body and kidney weights

The bw of rats in all groups were weighed at the beginning of treatment (1th day) and at the end of the experimental period (29th day) and recorded. Kidney tissues were quickly removed from the dissected rats, separated from the attached tissues, and the right and left kidney weights were recorded.

The relative weight of the kidney was expressed as g/100 g of the bw.

### Renal function markers

Renal function markers like uric acid, creatinine, and blood urea nitrogen (BUN) were analyzed spectrophotometrically in serum samples of rats with the Cobas c501 auto analyzer (Roche Diagnostics GmbH, Mannheim, Germany). Uric acid level was determined using enzymatic colorimetric test using the commercial “Cobas UA2, uric acid ver. 2” kit (Roche Diagnostics GmbH, Mannheim, Germany). Serum creatinine levels were measured with a kinetic colorimetric assay based on the Jaffé method with the “Cobas, CREJ 2” kit (Roche Diagnostics GmbH, Mannheim, Germany). Serum BUN was performed with “Cobas Ureal” kit (Roche Diagnostics GmbH, Mannheim, Germany) using a kinetic test with urease and glutamate dehydrogenase.

### Renal oxidative stress markers

Renal tissue homogenates were prepared using the Silent Crusher M homogenizer (Heidolph, Germany) and the tissue homogenates were centrifuged and supernatants were then separated. The obtained supernatants were used to determine the MDA levels and antioxidant enzyme activities. Renal biochemical analyses were performed spectrophotometrically using a spectrophotometer (Shimadzu UV 1700, Kyoto, Japan). The protein content in the renal tissue was measured in accordance with the Lowry et al.^25^

MDA level was measured as the lipid peroxidation index. The renal tissue MDA level was determined by reacting with TBA at the wavelength of 532 nm according to the method of Ohkawa et al.^26^ The activity is presented as nanomoles per milligram of protein.

The total superoxide dismutase (SOD) activity in renal supernatant was determined by analysis of the autoxidation and illumination of pyrogallol for 180 s at 440 nm.^27^ The activity is presented as units per milligram protein.

The renal catalase (CAT) activity was determined using the of Aebi method.^28^ The kidney tissue was diluted using Triton X-100. The enzyme activity was determined spectrophotometrically by measuring the rate of degradation of hydrogen peroxide at 240 nm. The activity is presented as millimole per milligram protein.

The glutathione peroxidase (GPx) activity in renal supernatant was determined as stated by the protocol of Paglia and Valentine,^29^ using H_2_O_2_ as the substrate. The reaction was investigated indirectly as the oxidation rate of NADPH for 3 min at 240 nm. The activity is presented as nanomoles per milligram of protein.

Glutathione-S-transferase (GST) activity was measured spectrophotometrically by using CDNB as a substrate in the presence of a cofactor glutathione by following the procedure as explained by Habig et al.^30^ The activity is presented as micromoles per milligram of protein.

### Histopathological study

For light microscopic analysis, the kidneys were fixed in 10% formaldehyde solution, treated with graded alcohol concentrations for dehydrate, cleared with xylene, and embedded in paraffin. The sections with 4–6 μm thicknesses obtained from renal tissue blocks were stained with hematoxylin and counter stained with eosin. Images of the sections were taken using a ToupCam XCAM 1080PHB camera mounted on an Olympus CX43 microscope. Kidney histopathological changes caused by exposure to fipronil in rats were evaluated using scale of none (0), mild (1), moderate (2), and severe (3) damage.

### Statistics

All statistical calculations were conducted using the PASW Statistics 18.0 software (SPSS, Inc., USA). Statistical differences among the application groups for biochemical parameters and incidence of histopathological changes were analyzed using 1-way ANOVA followed by Tukey’s post hoc test for multiple comparisons. All findings were represented as the means ± standard deviation (S.D.) and statistically significant was accepted at *P* < 0.05.

## Results

Mortality was not observed during the 28-day experimental period in rats. In the present study, no significant differences were found in any analyzed parameters between the group given corn oil and groups treated with curcumin and quercetin. Furthermore, the corn oil-treated group was used as a control group for this study.

### Body and kidney weights

At the end of 28 days, the initial and final bw and bw gain did not show any significant differences, when fipronil, fipronil plus quercetin, fipronil plus curcumin, and fipronil plus quercetin plus curcumin groups were compared with the control group (data not shown).

At the same time, there were no meaningful changes among the control rat and all fipronil-treated rats in terms of relative and absolute kidney weights (data not shown).

### Renal function markers

**Fig. 2:**
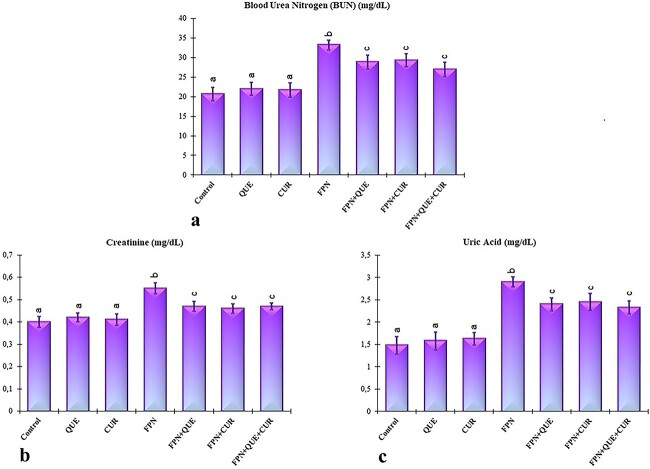
Effects of fipronil (FPN), quercetin (QUE), and curcumin (CUR) on BUN a), creatinine b), and uric acid c) levels in serum of rats. Each column is expressed as means ± S.D. (*n* = 6). The columns carrying different letters are statistically significantly different (*P* < 0.05).

Figure 2a–c represents the result of fipronil treatment on BUN, creatinine, and uric acid levels in serum. Data showed that fipronil exposure led to a significant increase in the serum levels of creatinine, BUN, and uric acid compared with the control group. Contrariwise, treatment with quercetin and/or curcumin substantially reduced the serum levels of BUN, creatinine, and uric acid compared with the only fipronil-treated group (Fig. 2a–c; *P* < 0.05).

### Renal oxidative stress markers

Oxidative stress was determined by assessing the level of MDA (an end-product lipid peroxidation) and the activities of the main enzymatic antioxidants (SOD, CAT, GPx, and GST) in renal tissue.

**Fig. 3:**
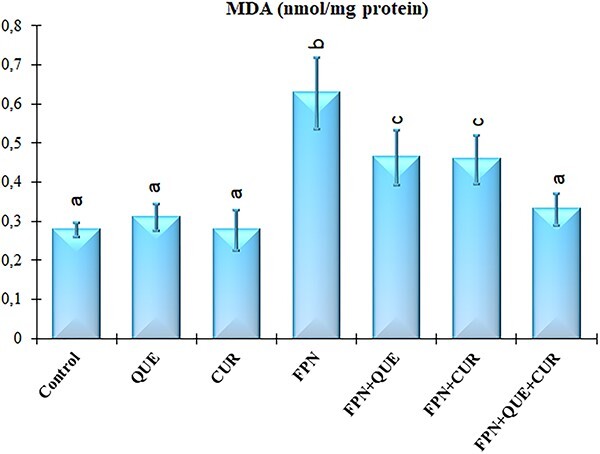
Effects of fipronil (FPN), quercetin (QUE), and curcumin (CUR) on MDA level in kidney tissues of rats. Each column is expressed as means ± S.D. (*n* = 6). The columns carrying different letters are statistically significantly different (*P* < .05).

MDA level produced was significantly high in kidney tissue of rats in fipronil-, fipronil plus quercetin-, and fipronil plus curcumin-treated groups compared with the control group (Fig. 3: *P* < 0.05). MDA level decreased when fipronil plus quercetin and fipronil plus curcumin groups were compared with the only fipronil-treated group. However, MDA levels in kidney tissue of rats in fipronil plus quercetin plus curcumin-treated group were not significantly different from the control (Fig. 3; *P* < 0.05).

**Fig. 4:**
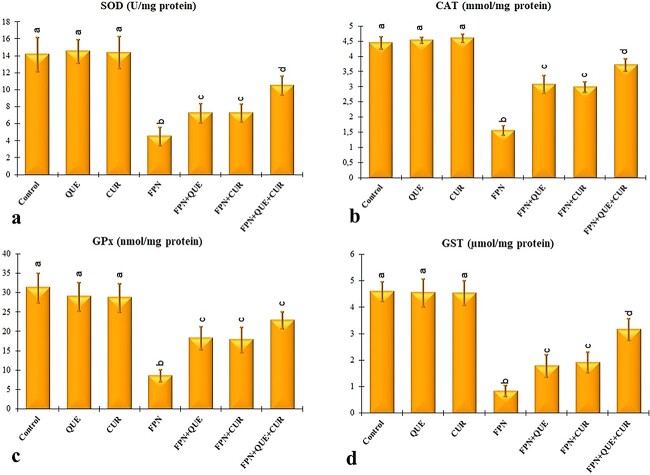
Effects of fipronil (FPN), quercetin (QUE), and curcumin (CUR) on renal SOD a), CAT b), GPx c), and GST d) activities in the kidney tissues of rats. Each column is expressed as means ± S.D. (*n* = 6). The columns carrying different letters are statistically significantly different (*P* < 0.05).

CAT, SOD, GST, and GPx activities in all fipronil-treated groups were significantly lower than those the control group (Fig. 4a–d; *P* < 0.05). Also, there was a statistically important increase in CAT, SOD, GST, and GPx activities when fipronil plus quercetin, fipronil plus curcumin, and fipronil plus quercetin plus curcumin groups were compared with the only with the fipronil group (Fig. 4a–d; *P* < 0.05). Moreover, the combined application of the 2 antioxidants significantly resulted in higher SOD, CAT, and GST enzyme activities compared with the individual application (Fig. 4a–d; *P* < 0.05).

### Renal histopathological analysis

The histopathological changes are graded and summarized in Table 1.

**Table 1:** The scores of the histological changes in kidney tissue of fipronil, quercetin, and curcumin exposure of rat.

Groups	Parameters					
	Inflammatory cell infiltration	Tubular degeneration	Glomerular degeneration	Glomerular atrophy	Edema	Congestion
Control	0^a^	0^a^	0^a^	0^a^	0^a^	0^a^
QUE	0^a^	0^a^	0^a^	0^a^	0^a^	0^a^
CUR	0^a^	0^a^	0a	0^a^	0^a^	0^a^
FPN	2.83 ± .41^b^	2.82 ± .41^b^	2.83 ± .41^b^	1.33 ± .52^b^	2.67 ± .52^b^	2.17 ± .41^b^
FPN + QUE	1.83 ± .41^c^	1.83 ± .41^c^	1.33 ± .52^c^	0^a^	1.17 ± .75^c^	1.17 ± .75^c^
FPN + CUR	1.33 ± .52^c^	2.00 ± .63^c^	1.33 ± .52^c^	0^a^	1.33 ± .52^c^	1.17 ± .75^c^
FPN + QU + CUR	0^a^	1.17 ± .41^d^	1.17 ± .41^c^	0^a^	1.00 ± .63^c^	1.00 ± .63^c^

*FPN Fipronil; QUE Quercetin; CUR Curcumin.*

Each data are expressed as means ± S.D.

Values with different letters in each column are statistically significantly different (*P* < 0.05).

**Fig. 5:**
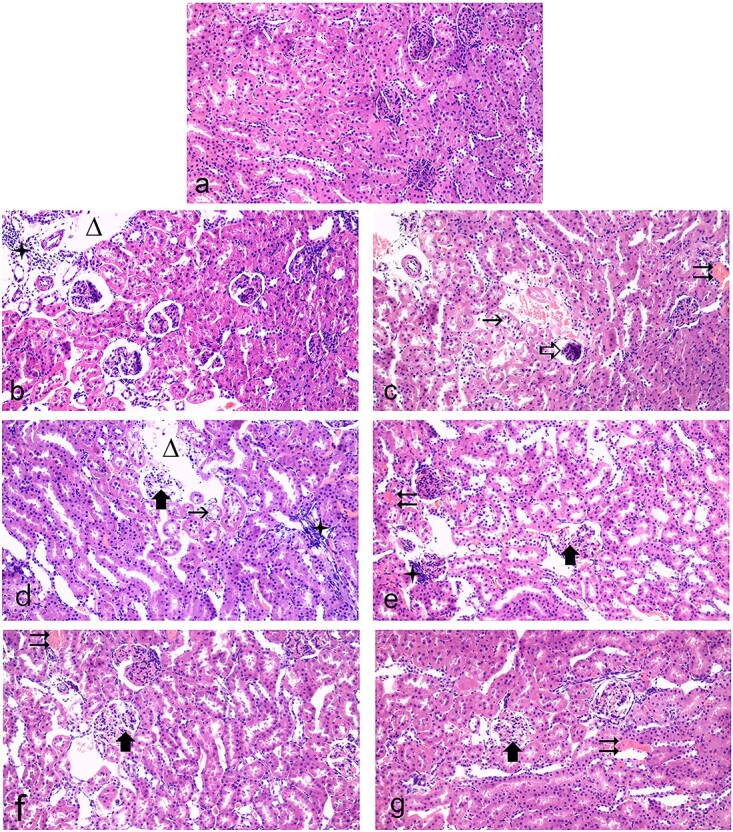
Histological evaluation of rat kidney tissues in H&E stained sections (×200). A representative photomicrograph showing the normal histological structure of kidney tissue in control rats, H&E. b–d) Kidney sections of fipronil-treated rats: tubular (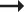) and glomerular degeneration (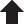), inflammatory cell infiltration (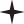), edema (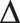), congestion (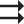), and glomerular atrophy (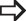). e) Kidney section of fipronil plus quercetin-treated rats: glomerular degeneration (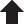) and congestion (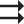). f) Kidney section of fipronil plus curcumin-treated rats: glomerular degeneration (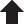) and congestion (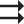). g) Kidney sections of fipronil plus quercetin plus curcumin-treated rats: glomerular degeneration (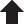) and congestion (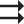).

The renal tissues of control, quercetin, and curcumin groups were observed the typical histological architectures with normal renal tubules and renal corpuscles (Fig. 5a). Inflammatory cell infiltration, tubular and glomerular degeneration, congestion, glomerular atrophy, and edema were detected in the kidney tissue of rats treated with fipronil (Fig. 5b–d).

Coadministration of quercetin or curcumin with fipronil for 28 days resulted in marked attenuation of the inflammatory cell infiltration, congestion, edema, tubular, and glomerular degeneration (Fig. 5e–f and Table 1). Concomitant administration of the quercetin and curcumin combination with fipronil resulted in a significant reduction in congestion, edema, tubular, and glomerular degeneration (*P* < .05), and no inflammatory cell infiltration was observed (Fig. 5g and Table 1). Furthermore, glomerular atrophy was not observed in the renal tissue the groups in which quercetin and curcumin were administered individually or in combination. Histopathologically, coadministration of quercetin and curcumin resulted in a significant reduction in renal tubular degeneration compared with individual administrations (Table 1; *P* < 0.05). Administration of quercetin and curcumin individually or in combination to rats treated with fipronil attenuated the histopathological changes in kidney tissue.

## Discussion

Fipronil is a fairly new phenylpyrazole insecticide that is widely used in many fields such as agriculture, veterinary medicine, and public health.^31^ Fipronil or its metabolites cause neurotoxicity by suppressing the inhibitory effect of gamma aminobutyric acid (GABA) by targeting chloride channels regulated by GABAA.^32^ In addition to this, fipronil causes toxicity in many tissues and organs through oxidative stress because of excessive accumulation of reactive oxygen species (ROS) in cells and disruption of antioxidant and oxidant system balance.^33^

In this study, it was assessed the possible protective influence of quercetin and curcumin, individually or in combination, against the nephrotoxic effects induced by fipronil administered orally at 1/25th LD_50_ for 28 days on rat renal tissue.

Evaluation of bw in experimental toxicological studies can be one of the indicator of the general health status of animals. Moreover, organ weight alterations have been accepted as an indicator of structural and functional changes caused by toxic substances in organ toxicity.^34^ In this study, no significant change was determined in bw and kidney weight of rats compared with the control group because of the administration of subacute fipronil at a dose of 3.88 mg/kg. Consistent with the present findings, Badgujar et al.^35^ reported that there was no significant effect of exposure fipronil on body and organ weights in mice. These results may be because the toxic effect of fipronil is dependent on the application time and/or dose.

The kidney is one of the main organs of the body performing vital functions to regulating blood pressure, maintaining hemostasis, participates in detoxification and waste excretion.^36^ In particular, renal excretion is the principal route of elimination of many xenobiotics, drugs, and their metabolites. As highlighted in previous studies, the kidneys are susceptible to the toxicity of various environmental pollutants such as pesticides, heavy metals, because of the high blood volume in the kidneys. It has been emphasized in many previous studies that insecticide exposure changes in renal functions and causes nephrotoxicity.^37–39^ BUN, creatinine, and uric acid are waste products of the metabolism that are mainly eliminated by the kidneys.^40^ So, they are the most sensitive biomarkers of nephrotoxicity used in the monitoring of kidney dysfunction.^41^ In this study, fipronil-induced renal dysfunction was manifested by an elevation in serum creatinine, BUN, and uric acid levels. In particular, serum creatinine and BUN levels are the commonly used as the main markers in the clinical evaluation of glomerular filtration function.^42^ BUN is the principal nitrogen-containing metabolic product of protein catabolism, which serves as a biochemical marker of renal function, renal damage, and evaluation of the function of the nephrons.^43^ Creatinine, a nonprotein nitrogenous substance, is a metabolic product formed from phosphocreatine and creatinine during muscle metabolism and is excreted in the urine.^43^ Since creatinine is almost completely filtered by the glomeruli, it is a more specific marker of impaired glomerular filtration than BUN.^32^ The elevated creatinine and BUN levels in serum indicate the reduced ability of the kidney to filter these waste substances from the blood and excrete them in the urine. These alterations in the renal functions may be attributed to the ability of fipronil to induce kidney damages. Uric acid, nucleotide metabolism, or the end product of purine metabolism, generated by the liver and a significant amount is excreted in the urine through the kidneys.^44^ Recently, it has been emphasized that uric acid, which is found at low levels in plasma, is a powerful endogenous antioxidant and plays an active role in many biological mechanisms.^45^ Increased uric acid levels may result because of decreased urate excretion by the kidneys but also from endogenous overproduction because of increased purine degradation or a combination of both.^46^ However, uric acid, an endogenous antioxidant, may be elevated in response to increased free radicals caused by fipronil. In this work, the changes in all markers of renal function examined are confirmed by the histological findings in our study. However, our results agree with previous studies reporting changes in renal function markers in serum following administration of fipronil.^7^^,^^47^^,^^48^ Based on these data, we can conclude that glomerular filtration and the renal tubular reabsorption functions are impaired in relation to glomerular and tubular damage in the renal tissues because of exposure to fipronil.

One of the important mechanisms that play a role in fipronil’s toxicity in organs is that it or its metabolites cause oxidative stress. Oxidative stress is induced by excessive ROS accumulation and leads to tissue damage. Previous studies have shown that increased ROS production in fipronil-induced toxicity plays a pivotal role in the development of toxicity in various organs and tissues such as the kidney,^32^ liver,^8^ and testis^49^ in experimental animals. Additionally, it was emphasized that increased ROS production may lead to changes in some organ histological structures and serum biomarkers.^50^ The results obtained from this study revealed that exposure to fipronil caused oxidative stress because of significantly decreasing CAT, SOD, GST, and GPx enzyme activities, significantly increasing MDA level. The changes in oxidative stress parameters in this study are consistent with the results obtained in kidney function tests and histological examinations of kidney tissue. The elevation of the lipid peroxidation marker MDA in the kidney indicates injury of cell membranes by ROS associated with the inability of the cellular enzymatic and nonenzymatic antioxidant systems to detoxicate the free radicals in this organ. Simultaneously, the MDA level, which increases the toxicity induced by xenobiotics, reacts with DNA, RNA, and proteins in cells and causes kidney tissue damage. Enzymatic antioxidizing agents, including SOD, CAT, GST, and GPx, effectively detoxify ROS and prevent oxidative stress in cells. Among the antioxidant enzymes, SOD converts superoxide ions, which are formed as by-products during oxidative stress to H_2_O_2_^51^, whereas CAT plays a role in the conversion of H_2_O_2_ to H_2_O and O_2_ by inhibiting the formation of OH.^52^ GPx plays a role in reducing lipid peroxides and H_2_O_2_ to alleviate oxidative stress.^53^ GST occupies a key position in the detoxification process by catalyzing the conjugation of glutathione to xenobiotic substrates.^54^ The present study showed that the administration of fipronil caused oxidative stress in the kidney tissue of rats by causing a dramatic decrease in enzyme activities associated with antioxidant defense mechanisms. Similar results have been confirmed by previous studies on tissues such as kidney,^4^ liver,^8^ brain^55^ after exposure to fipronil. The reduction of endogenous antioxidants in the renal tissue of rats exposed to fipronil could be because of excess generation of superoxide radicals, which are rapidly converted into H_2_O_2_ by SOD and to water by CAT and GPx.^55^ This view has been confirmed by previous research reporting that applications of fipronil cause excessive production of ROS such as superoxide anions and hydroxyl radical in cells.^56^^,^^57^

The evaluation of histopathological changes in tissues and organs is important in toxicity studies related to environmental pollutants. In this study, the subacute renal toxic effect of fipronil was demonstrated by elevated serum uric acid, creatinine, and BUN levels, the changes in oxidative stress parameters, as well as histopathological changes such as glomerular and tubular degeneration, glomerular atrophy, edema, congestion, and mononuclear cell infiltration. These results showed clear changes in the general condition of the kidney tissue in response to fipronil. These histological changes may be because of increased renal MDA levels and reduced antioxidative enzyme activities in the renal tissue in fipronil-treated rats. Similar histopathological alterations in renal tissue were also reported by other researchers following insecticide treatments.^7^^,^^32^

Natural antioxidants,^2^ which can protect cells against oxidative stress caused by environmental pollutants, are involved in potential antioxidant therapy.^55^ Quercetin and curcumin are natural plant-derived bioactive molecules with strong antioxidant properties.^58^ In this study, it was observed that the supplementation of quercetin or curcumin increased antioxidant capacity by increasing CAT, SOD, GPx, and GST activities and reduced the oxidant status by decreasing MDA levels in renal tissue. However, the coadministration of quercetin and curcumin significantly improved the antioxidant enzymes SOD, CAT, and GST activities and MDA levels in the kidney tissue of rats compared with the groups in which they were administered individually. According to these data obtained from this study, it can be said that quercetin and curcumin have a synergistic effect. Currently, there are various in vitro, in vivo, and clinical studies that demonstrate the potential of curcumin as an antioxidant, anti-inflammatory, anticancer, antiviral, and antidiabetic compound.^59^ Similarly, it has been stated in various studies that quercetin has various pharmacological effects such as, antioxidant, anti-inflammatory, and anticarcinogen.^60^^,^^61^ The antioxidant activity of curcumin against free radicals has been attributed to the presence of methoxy and phenolic groups on the phenyl ring and 1,3-diketo group in its chemical structure.^62^ It has been reported that curcumin inhibits hydroxyl radical, nitrogen dioxide, superoxide radical, and singlet oxygen.^63^ Additionally, curcumin has cytoprotective effects against oxidative damage by increasing nuclear factor erythroid 2-related factor 2 (Nrf2) activation and reducing ROS generation.^64^ Nrf2 is a transcription factor that is responsible for regulating the expression of genes encoding many of the phase II detoxification and antioxidant enzymes, sensitive to oxidative stress and of great importance for maintaining cell homeostasis.^65^ Additionally, quercetin, like curcumin, shows antioxidant activity by affecting the Nrf2 signaling pathway.^66^ The chemical structure of quercetin contains many phenolic hydroxyl groups that show strong antioxidant properties.^67^ Also, in this study, the administration of quercetin and/or curcumin also improved the kidney function markers that fipronil caused changes. Cai et al.^68^ reported that curcumin has a protective effect against acute kidney injury and that curcumin can increase Nrf2 expression to inhibit cellular oxidative stress and protect kidney function from kidney damage. Our results revealed that quercetin and/or curcumin eased the oxidative stress parameters, renal function markers, and histopathological changes, induced by fipronil in the rat renal tissue. Our proposed mechanism for such effects in the present study is the ability of quercetin and curcumin to reduce lipid peroxidation and enhance cellular antioxidant defense through the activation of the Nrf2 signaling pathway associated with the expression of antioxidant enzymes. Thus, the oxidative damage caused by fipronil in the kidney tissue decreased. Decreased oxidative stress with antioxidant applications improved kidney function and alleviation of histopathological changes. Similarly, Uzunhisarcıklı et al.^8^ reported that the administration of curcumin and quercetin provided improvement in structural and functional disorders in liver tissue caused by fipronil.

## Conclusion

Considering the results obtained from the present study, fipronil causes renal damage by way of oxidative stress. Decreased activities of antioxidant enzymes and increased levels of lipid peroxidation reveal that fipronil disrupts the prooxidant/antioxidant balance and causes oxidative stress by increasing ROS formation. Because of oxidative stress, structural and functional disorders were detected in the kidney tissue. However, the supplementation of quercetin and/or curcumin via their free radical scavenging and antioxidant characteristics proved to be helpful in decreasing the fipronil-induced nephrotoxicity.

## Author contributions

All authors contributed at every stage of the study. Meltem Uzunhisarcikli, Fatma G. Apaydin, Hatice Bas, and Yusuf Kalender contributed to the design of the study, the realization of animal experiments, analysis and interpretation of results, and the writing of the manuscript. Meltem Uzunhisarcikli was responsible for editing and revising the whole manuscript. All authors read and approved the final manuscript.

## Data Availability

All data analyzed regarding renal toxicity during this study were included in the manuscript.

## References

[1] Chagnon M , Kreutzweiser D, Mitchell EAD, Morrissey CA, Noome DA, Van der Sluijs JP. Risks of large-scale use of systemic insecticides to ecosystem functioning and services. Environ Sci Pollut Res. 2015:22(1):119–134. 10.1007/s11356-014-3277-x. PMC4284381 25035052

[2] Chaguri JL , Godinho AF, Horta DF, Horta DF, Goncalves-Rizzi VH, Possomato-Vieira VH, Possomato-Viera JS, Nascimento RA, Dias-Junior CA. Exposure to fipronil elevates systolic blood pressure and disturbs related biomarkers in plasma of rats. Environ Toxicol Pharmacol. 2016:42:63–68. 10.1016/j.etap.2015.12.020. 26773360

[3] Badgujar PC , Pawar NN, Chandratre GA, Telang AG, Sharma AK. Fipronil induced oxidative stress in kidney and brain of mice: protective effect of vitamin E and vitamin C. Pestic Biochem Physiol. 2015:118:10–18. 10.1016/j.pestbp.2014.10.013. 25752424

[4] Abdel-Daim MM , Abdeen A. Protective effects of rosuvastatin and vitamin E against fipronil-mediated oxidative damage and apoptosis in rat liver and kidney. Food Chem Toxicol. 2018:114:69–77. 10.1016/j.fct.2018.01.055. 29432839

[5] Bharatiya R , Bratzu J, Lobina C, Corda G, Cocco C, Deurwaerdere P, Argiolas A, Melis MR, Sanna F. The pesticide fipronil injected into the substantia nigra of male rats decreases striatal dopamine content: A neurochemical, immunohistochemical and behavioral study. Behav Brain Res. 2020:384:1112562. 10.1016/j.bbr.2020.112562. 32070689

[6] Leghait J , Gayrard V, Picard-Hagen N, Camp M, Perdu E, Toutain PL, Viguié C. Fipronil-induced disruption of thyroid function in rats is mediated by increased total and free thyroxine clearances concomitantly to increased activity of hepatic enzymes. Toxicology. 2009:255(1–2):38–44. 10.1016/j.tox.2008.09.026. 18977275

[7] Mossa AH , Swelam ES, Mohafrash SMM. Sub-chronic exposure to fipronil induced oxidative stress, biochemical and histopathological changes in the liver and kidney of male albino rats. Toxicol Rep. 2015:2:775–784. 10.1016/j.toxrep.2015.02.009. 28962413 PMC5598362

[8] Uzunhisarcikli M , Apaydin FG, Bas H, Kalender Y. Hepatoprotective effects of quercetin and curcumin against fipronil-induced hepatic injury in rats. Fresenius Environ Bull. 2021:30(7A):9309–9321.

[9] Bisht K , Wagner KH, Bulmer AC. Curcumin, resveratrol and flavonoids as anti-inflammatory, cyto- and DNA-protective dietary compounds. Toxicology. 2010:278(1):88–100. 10.1016/j.tox.2009.11.008. 19903510

[10] Kalender S , Apaydın FG, Kalender Y. Testicular toxicity of orally administrated bisphenol a in rats and protective role of taurine and curcumin. Pak J Pharm Sci. 2019:32(3):1043–1047. 31278718

[11] Seydi E , Mehrpouya L, Sadeghi H, Rahimi S, Pourahmad J. Luteolin attenuates fipronil-induced neurotoxicity through reduction of the ROS-mediated oxidative stress in rat brain mitochondria. Pestic Biochem Physiol. 2021:173:104785. 10.1016/j.pestbp.2021.104785. 33771263

[12] Ali N , AlAsmari AF, Imam F, Ahmed MZ, Alqahtani F, Alharbi M, AlSwayyed M, Alasmari F, Alasmari M, Alshammari A, et al. Protective effect of diosmin against doxorubicin-induced nephrotoxicity. Saudi J Biol Sci. 2021:28(8):4375–4383. 10.1016/j.sjbs.2021.04.030. 34354422 PMC8324953

[13] Bhattacharjee A , Kulkarni VH, Chakraborty M, Habbu PV, Ray A. Ellagic acid restored lead-induced nephrotoxicity by anti-inflammatory, anti-apoptotic and free radical scavenging activities. Heliyon. 2021:7(1):e05921. 10.1016/j.heliyon.2021.e05921. 33490681 PMC7809373

[14] Apaydin FG , Aslanturk A, Uzunhisarcikli M, Bas H, Kalender S, Kalender Y. Histopathological and biochemical studies on the effect of curcumin and taurine against bisphenol a toxicity in male rats. Environ Sci Pollut Res. 2019:26(12):12302–12310. 10.1007/s11356-019-04578-4. 30840252

[15] Oyovwi MO , Ben-Azu B, Tesi EP, Oyeleke AA, Uruaka CI, Rotu RA, Aya-Ebi EO. Repeated endosulfan exposure induces changes in neurochemicals, decreases ATPase transmembrane ionic-pumps, and increased oxidative/nitrosative stress in the brains of rats: reversal by quercetin. Pestic Biochem Physiol. 2021:175:104833. 10.1016/j.pestbp.2021.104833. 33993958

[16] Bagheri A , Ebrahimpour S, Nourbakhsh N, Talebi S, Esmaeili A. Protective effect of quercetin on alteration of antioxidant genes expression and histological changes in the dental pulp of the streptozotocin-diabetic rats. Arch Oral Biol. 2021:215:105088. 10.1016/j.archoralbio.2021.105088. 33640557

[17] Zeng X , Du Z, Ding X, Jiang W. Protective effects of dietary flavonoids against pesticide-induced toxicity: a review. Trends Food Sci Technol. 2021:109:271–279. 10.1016/j.tifs.2021.01.046.

[18] Yadav RS , Shukla RK, Sankhwar ML, Patel DK, Ansari RW, Pant AB, Islam F, Khanna VK. Neuroprotective effect of curcumin in arsenic-induced neurotoxicity in rats. Neurotoxicology. 2010:31(5):533–539. 10.1016/j.neuro.2010.05.001. 20466022

[19] Wang GL , Fu YC, Xu WC, Feng YQ, Fang SR, Zhou XH. Resveratrol inhibits the expression of SREBP1 in cell model of steatosis via Sirt1–FOXO1 signaling pathway. Biochem Biophys Res Commun. 2009:380(3):644–649. 10.1016/j.bbrc.2009.01.163. 19285015

[20] Sengottuvelan M , Deeptha K, Nalini N. Resveratrol ameliorates DNA damage, prooxidant and antioxidant imbalance in 1,2-dimethylhydrazine induced rat colon carcinogenesis. Chem Biol Interact. 2009:181(2):193–201. 10.1016/j.cbi.2009.06.004. 19523937

[21] Tingle CC , Rother JA, Dewhurst CF, Lauer S, King WJ. Fipronil: environmental fate, ecotoxicology, and human health concerns. Rev Environ Contam Toxicol. 2003:176:1–66. 978-1-4899-7283-5_1. 12442503 10.1007/978-1-4899-7283-5_1

[22] Arya A , Al-Obaidi MMJ, Shahid N, Noordin MIB, Looi CY, Wong WF, Khaing SL, Mustafa MR. Synergistic effect of quercetin and quinic acid by alleviating structural degeneration in the liver, kidney and pancreas tissues of STZ-induced diabetic rats: a mechanistic study. Food Chem Toxicol. 2014:71:183–196. 10.1016/j.fct.2014.06.010. 24953551

[23] Lonare M , Kumar M, Raut S, More A, Doltade S, Badgujar P, Telang A. Evaluation of imidacloprid-induced neurotoxicity in male rats: a protective effect of curcumin. Neurochem Int. 2014:78:122–129. 10.1016/j.neuint.2014.09.004. 25261201

[24] Abdel-Wahhab MA , Aljawish A, El-Nekeety AA, Abdel-Aziem SH, Hassan NS. Chitosan nanoparticles plus quercetin suppress the oxidative stress, modulate DNA fragmentation and gene expression in the kidney of rats fed ochratoxin A-contaminated diet. Food Chem Toxicol. 2017:99:209–221. 10.1016/j.fct.2016.12.002. 27923682

[25] Lowry OH , Rosebrough NJ, Farr AL, Randall RJ. Protein measurement with the Folin phenol reagent. J Biol Chem. 1951:19(1):265–275. 14907713

[26] Ohkawa H , Ohishi N, Yagi K. Assay of lipid peroxidation in animal tissues by thiobarbituric acid reaction. Anal Biochem. 1979:95(2):351–358. 10.1016/0003-2697(79)90738-3. 36810

[27] Marklund S , Marklund G. Involvement of the superoxide anion radical in the autoxidation of pyrogallol and a convenient assay for superoxide dismutase. Eur J Biochem. 1974:47(3):469–474. 10.1111/j.1432-1033.1974.tb03714.x. 4215654

[28] Aebi H . Catalase in vitro. Methods Enzymol. 1984:105:121–126. 10.1016/s0076-6879(84)05016-3. 6727660

[29] Paglia DE , Valentine WN. Studies on the quantitative and qualitative characterization of erythrocytes glutathione peroxidase. J Lab Clin Med. 1967:70(1):158–165. 6066618

[30] Habig WH , Pabst MJ, Jakoby WB. Glutathione S-transferases: the first enzymatic step in mercapturic acid formation. J Biol Chem. 1974:249(22):7130–7139. 10.1016/S0021-9258(19)42083-8. 4436300

[31] Awad MA , Ahmed ZSO, AbuBakr HO, El-Fattah AA, Elbargeesy H, Moussa MHG. Oxidative stress, apoptosis and histopathological alterations in brain stem and diencephalon induced by subacute exposure to fipronil in albino rats. Environ Sci Pollut Res. 2022:29(1):936–948. 10.1007/s11356-021-15537-3. 34345985

[32] Abou-Zeid SM , Tahoun EA, HO AB. Ameliorative effects of jojoba oil on fipronil-induced hepatorenal and neuro-toxicity: the antioxidant status and apoptotic markers expression in rats. Environ Sci Pollut Res. 2021:28(20):25959–25971. 10.1007/s11356-020-12083-2. 33481197

[33] Das PC , Cao Y, Cherrington N, Hodgson E, Rose RL. Fipronil induces CYP isoforms and cytotoxicity in human hepatocytes. Chem Biol Interact. 2006:164(3):200–214. 10.1016/j.cbi.2006.09.013. 17084830

[34] Michael B , Yano B, Sellers RS, Perry R, Morton D, Roome N, Johnson JK, Schafer K. Evaluation of organ weights for rodent and non-rodent toxicity studies: a review of regulatory guidelines and a survey of current practices. Toxicol Pathol. 2007:35(5):742–750. 10.1080/01926230701595292. 17849357

[35] Badgujar PC , Chandratre GA, Pawar NN, Telang AG, Kurade NP. Fipronil induced oxidative stress involves alterations in SOD1 and catalase gene expression in male mice liver: protection by vitamins E and C. Environ Toxicol. 2016:31(9):1147–1158. 10.1002/tox.22125. 25721553

[36] Mazher KM , Ahmed OM, Sayed HA, Nabil TM. The role of bone marrow-derived mesenchymal stromal cells and hesperidin in ameliorating nephrotoxicity ınduced by cisplatin in male Wistar rats. Int J Mol Cell Med. 2021:10(2):133–146. 10.22088/IJMCM.BUMS.10.2.133. 34703797 PMC8496246

[37] Kalender S , Kalender Y, Durak D, Ogutcu A, Uzunhisarcikli M, Cevrimli BS, Yildirim M. Methyl parathion induced nephrotoxicity in male rats and protective role of vitamins C and E. Pestic Biochem Physiol. 2007:88(2):213–218. 10.1016/j.pestbp.2006.11.007.

[38] Adiguzel C , Kalender Y. Bendiocarb-induced nephrotoxicity in rats and the protective role of vitamins C and E. Environ Sci Pollut Res. 2020:27(6):6449–6458. 10.1007/s11356-019-07260-x. 31873894

[39] Aslanturk A , Kalender Y. Methomyl-induced nephrotoxicity and protective effect of curcumin in male rats. Toxicol Res. 2021:10(5):1003–1012. 10.1093/toxres/tfab080. PMC8557666 34733485

[40] Poornima V , Thangalakshmi S. Biochemical screening of diabetic nephropathy patients in mani hospital. Thiruthuraipoondi. J Chem Pharm Res. 2015:7(10):540–545.

[41] Sultana S , Verma K, Khan R. Nephroprotective efficacy of chrysin against cisplatin-induced toxicity via attenuation of oxidative stress. J Pharm Pharmacol. 2012:64(6):872–881. 10.1111/j.2042-7158.2012.01470.x. 22571266

[42] Kong J , He T, Liu C, Huang J. Multi modular toxicity assessment of nephrotoxicity in podophyllotoxin exposure rats on account of toxicological evidence chain (TEC) concept. Ecotoxicol Environ Saf. 2022:231:113157. 10.1016/j.ecoenv.2021.113157. 35026582

[43] Ezebuiro I , Ododo A, Apugo UI. Hepato-renal activities of hydro-methanol leaf extract of cnidoscolus aconitifoliusin adult male Wistar rats. J Drug Deliv Ther. 2021:11(4):5–9. 10.22270/jddt.v11i4.4870.

[44] Zou F , Zhao X. Wang F (2021) A review on the fruit components affecting uric acid level and their underlying mechanisms. J Food Biochem. 2021:45(10):e13911. 10.1111/jfbc.13911. 34426969

[45] Jiang LL , Gong X, Ji MY, Wang CC, Wang JH, Li MH. Bioactive compounds from plant-based functional foods: a promising choice for the prevention and management of hyperuricemia. Foods. 2020:9(8):973. 10.3390/foods9080973. 32717824 PMC7466221

[46] Lee TH , Chen JJ, Wu CY, Yang CW, Yang HY. Hyperuricemia and progression of chronic kidney disease: a review from physiology and pathogenesis to the role of urate-lowering therapy. Diagnostics. 2021:11(9):1674. 10.3390/diagnostics11091674. 34574015 PMC8466342

[47] Abd Eldaim MAA , Abd El Latif AS, Hassan A, El-Borai NM. Ginseng attenuates fipronil-induced hepatorenal toxicity via its antioxidant, anti-apoptotic, and anti-inflammatory activities in rats. Environ Sci Pollut Res. 2020:27(36):45008–45017. 10.1007/s11356-020-10306-0. 32772290

[48] Sakr S , Hamed A, Atef M. Betanin ameliorates fipronil-induced nephrotoxicity via activation of Nrf2-HO-1/NQO-1 pathway in albino rat model. Toxicol Res. 2022:11(6):975–986. 10.1093/toxres/tfac076. PMC9773064 36569480

[49] Tohamy HG , El-Kazaz SE, Alotaibi SS, Ibrahiem HS, Shukry M, Dawood MAO. Ameliorative effects of boswellic acid on fipronil-ınduced toxicity: antioxidant state, apoptotic markers, and testicular steroidogenic expression in male rats. Animals. 2021:11(5):1302. 10.3390/ani11051302. 33946602 PMC8147226

[50] Mustafa HN , El Awdan SA, Hegazy GA, Jaleel A. Prophylactic role of coenzyme Q10 and Cynara scolymus L on doxorubicin-induced toxicity in rats: biochemical and immunohistochemical study. Indian J Pharmacol. 2015:15(6):649–656. 10.4103/0253-7613.169588. PMC4689020 26729958

[51] Kheradmand A , Alirezaei M, Birjandi M. Ghrelin promotes antioxidant enzyme activity and reduces lipid peroxidation in the rat ovary. Regul Pept. 2010:162(1–3):84–89. 10.1016/j.regpep.2010.02.008. 20171996

[52] Halliwell B . Free radicals, reactive oxygen species and human disease: a critical evaluation with special reference to atherosclerosis. Br J Exp Pathol. 1989:70(6):737–757. 2557883 PMC2040729

[53] Safhi MM . Nephroprotective effect of zingerone against CCl4-induced renal toxicity in swiss albino mice: molecular mechanism. Oxidative Med Cell Longev. 2018:2018:2474831. 10.1155/2018/2474831. PMC5831687 29636837

[54] Rizwan S , Naqshbandi A, Farooqui Z, Khan AA, Khan F. Protective effect of dietary flaxseed oil on arsenic-induced nephrotoxicity and oxidative damage in rat kidney. Food Chem Toxicol. 2014:68:99–107. 10.1016/j.fct.2014.03.011. 24642383

[55] Khalaf AA , Galal MK, Ibrahim MA, Abd Allah AA, Afify MM, Refaat R. The *Terminalia laxiflora* modulates the neurotoxicity induced by fipronil in male albino rats. Biosci Rep. 2019:39(3) BSR20181363. 10.1042/BSR20181363. PMC6395302 30777931

[56] Vidau C , Diogon M, Aufauvre J, Fontbonne R, Viguès B, Brunet JL, Texier C, Biron DG, Blot N, El Alaoui H, et al. Exposure to sublethal doses of fipronil and thiacloprid highly ıncreases mortality of honeybees previously ınfected by *Nosema ceranae*. PLoS One. 2011:6(6):e21550. 10.1371/journal.pone.0021550. 21738706 PMC3125288

[57] Wang XQ , Li YG, Zhong S, Zhang H, Wang XY, Qi PP, Xu H. Oxidative injury is involved in fipronil-induced G2/M phase arrest and apoptosis in *Spodoptera frugiperda* (Sf9) cell line. Pestic Biochem Physiol. 2013:105(2):122–130. 10.1016/j.pestbp.2012.12.008.

[58] Sadeghi-Ghadi Z , Vaezi A, Ahangarkani F, Ilkit M, Ebrahimnejad P, Badali H. Potent in vitro activity of curcumin and quercetin co-encapsulated in nanovesicles without hyaluronan against *Aspergillus* and *Candida* isolates. J Mycol Med. 2020:30(4):101014. 10.1016/j.mycmed.2020.101014. 32800427

[59] Fu YS , Chen TH, Weng L, Huang L, Lai D, Weng CF. Pharmacological properties and underlying mechanisms of curcumin and prospects in medicinal potential. Biomed Pharmacother. 2021:141:111888. 10.1016/j.biopha.2021.111888. 34237598

[60] Lesjak M , Beara I, Simin N, Pintac D, Majkić T, Bekvalac K, Orčić D, Mimica-Dukić. Antioxidant and anti-inflammatory activities of quercetin and its derivatives. J Funct Foods. 2018:40(1):68–75. 10.1016/j.jff.2017.10.047.

[61] Ezzati M , Yousefi B, Velaei K, Safa A. A review on anticancer properties of quercetin in breast cancer. Life Sci. 2020:248(9):117463. 10.1016/j.lfs.2020.117463. 32097663

[62] Priyadarsini KI , Maity DK, Naik GH, Kumar MS, Unnikrishnan MK, Satav JG, Mohan H. Role of phenolic O–H and methylene hydrogen on the free radical reactions and antioxidant activity of curcumin. Free Radic Biol Med. 2003:35(5):475–484. 10.1016/S0891-5849(03)00325-3. 12927597

[63] Laorodphun P , Cherngwelling R, Panya A, Arjinajarn P. Curcumin protects rats against gentamicin-induced nephrotoxicity by amelioration of oxidative stress, endoplasmic reticulum stress and apoptosis. Pharm Biol. 2022:60(1):491–500. 10.1080/13880209.2022.2037663. 35188833 PMC8865128

[64] Xie YL , Chu JG, Jian XM, Dong JZ, Wang LP, Li GX, Yang NB. Curcumin attenuates lipopolysaccharide/D-galactosamine-induced acute liver injury by activating Nrf2 nuclear translocation and inhibiting NF-kB activation. Biomed Pharmacother. 2017:91:70–77. 10.1016/j.biopha.2017.04.070. 28448872

[65] Lu Y , Wu S, Xiang B, Li L, Li Y. Curcumin attenuates oxaliplatin-induced liver injury and oxidative stress by activating the Nrf2 pathway. Drug Des Devel Ther. 2020:14:73–85. 10.2147/DDDT.S224318. PMC6956999 32021093

[66] Ashari S , Karami M, Shokrzadeh M, Bagheri A, Ghandadi M, Ranaee M, Dashti A, Mohammadi H. Quercetin ameliorates Di (2-ethylhexyl) phthalate-induced nephrotoxicity by inhibiting NF-κB signaling pathway. Toxicol Res. 2022:11(2):272–285. 10.1093/toxres/tfac006. PMC9052324 35510228

[67] Tieppo J , Cuevas MJ, Vercelino R, Tunon MJ, Marroni NP, González-Gallego J. Quercetin administration ameliorates pulmonary complications of cirrhosis in rats. J Nutr. 2009:139(7):1339–1346. 10.3945/jn.109.105353. 19494027

[68] Cai Y , Huang C, Zhou M, Xu S, Xie Y, Gao S, Yang Y, Deng Z, Zhang L, Shu J, et al. Role of curcumin in the treatment of acute kidney injury: research challenges and opportunities. Phytomedicine. 2022:104:154306. 10.1016/j.phymed.2022.154306. 35809376

